# Gender Medicine in Clinical Radiology Practice

**DOI:** 10.3390/jpm13020223

**Published:** 2023-01-27

**Authors:** Giuliana Giacobbe, Vincenza Granata, Piero Trovato, Roberta Fusco, Igino Simonetti, Federica De Muzio, Carmen Cutolo, Pierpaolo Palumbo, Alessandra Borgheresi, Federica Flammia, Diletta Cozzi, Michela Gabelloni, Francesca Grassi, Vittorio Miele, Antonio Barile, Andrea Giovagnoni, Nicoletta Gandolfo

**Affiliations:** 1 General and Emergency Radiology Department, “Antonio Cardarelli” Hospital, 80131 Naples, Italy; 2 Division of Radiology, Istituto Nazionale Tumori IRCCS Fondazione Pascale—IRCCS di Napoli, 80131 Naples, Italy; 3 Medical Oncology Division, Igea SpA, 80013 Naples, Italy; 4 Department of Medicine and Health Sciences “V. Tiberio”, University of Molise, 86100 Campobasso, Italy; 5 Department of Medicine, Surgery and Dentistry, University of Salerno, 84084 Salerno, Italy; 6 Department of Diagnostic Imaging, Area of Cardiovascular and Interventional Imaging, Abruzzo Health Unit 1, 67100 L’Aquila, Italy; 7 Italian Society of Medical and Interventional Radiology (SIRM), SIRM Foundation, 20122 Milan, Italy; 8 Department of Clinical, Special and Dental Sciences, University Politecnica delle Marche, 60126 Ancona, Italy; 9 Department of Radiology, University Hospital “Azienda Ospedaliera Universitaria delle Marche”, Via Conca 71, 60126 Ancona, Italy; 10 Department of Emergency Radiology, Careggi University Hospital, Largo Brambilla 3, 50134 Florence, Italy; 11 Department of Translational Research, Diagnostic and Interventional Radiology, University of Pisa, 56126 Pisa, Italy; 12 Division of Radiology, “Università degli Studi della Campania Luigi Vanvitelli”, 80138 Naples, Italy; 13 Department of Applied Clinical Sciences and Biotechnology, University of L’Aquila, 67100 L’Aquila, Italy; 14 Diagnostic Imaging Department, Villa Scassi Hospital-ASL 3, Corso Scassi 1, 16149 Genoa, Italy

**Keywords:** gender medicine, radiology, diagnosis, prognosis

## Abstract

Gender Medicine is rapidly emerging as a branch of medicine that studies how many diseases common to men and women differ in terms of prevention, clinical manifestations, diagnostic-therapeutic approach, prognosis, and psychological and social impact. Nowadays, the presentation and identification of many pathological conditions pose unique diagnostic challenges. However, women have always been paradoxically underestimated in epidemiological studies, drug trials, as well as clinical trials, so many clinical conditions affecting the female population are often underestimated and/or delayed and may result in inadequate clinical management. Knowing and valuing these differences in healthcare, thus taking into account individual variability, will make it possible to ensure that each individual receives the best care through the personalization of therapies, the guarantee of diagnostic-therapeutic pathways declined according to gender, as well as through the promotion of gender-specific prevention initiatives. This article aims to assess potential gender differences in clinical-radiological practice extracted from the literature and their impact on health and healthcare. Indeed, in this context, radiomics and radiogenomics are rapidly emerging as new frontiers of imaging in precision medicine. The development of clinical practice support tools supported by artificial intelligence allows through quantitative analysis to characterize tissues noninvasively with the ultimate goal of extracting directly from images indications of disease aggressiveness, prognosis, and therapeutic response. The integration of quantitative data with gene expression and patient clinical data, with the help of structured reporting as well, will in the near future give rise to decision support models for clinical practice that will hopefully improve diagnostic accuracy and prognostic power as well as ensure a more advanced level of precision medicine.

## 1. Introduction

In clinical-medical practice, drug trials, and scientific research, the topic of gender differences is fairly recent history.

However, a growing body of epidemiological, clinical, and experimental data increasingly indicates the existence of relevant differences in the onset, progression, and clinical manifestations of countless diseases common to men and women, as well as in the response to therapeutic treatments as well as in adverse events [1]. 

Gender medicine or, rather, gender-specific medicine is defined by the World Health Organization (WHO) as the study of how biological (defined by sex), socioeconomic, and cultural (defined by gender) differences influence the health and disease status of an individual and/or a group of individuals.

Underlying the introduction of this new referral model is an awareness of the need to tailor the entire diagnostic-therapeutic pathway to each individual, taking into account congenital gender differences. In fact, only a gender approach in clinical practice will make it possible to promote appropriateness and personalization of care [2,3]. 

Gender medicine, therefore, does not represent a separate branch of the medical area but an interdisciplinary dimension that, as such, must pervade all medical branches in order to study the influence of sex and gender on human physiology, pathophysiology, and pathology and, therefore, how diseases develop, what the symptoms are, and how prevention, diagnosis, and therapy are performed [4,5]. 

Nowadays, the presentation and identification of many diseases common to men and women pose unique diagnostic challenges. In addition, because of gender differences, many clinical conditions in the female population are underestimated and/or delayed and may result in poor clinical management [6,7]. 

The purpose of this article is to assess potential gender differences in clinical-radiology practice and their impact on health and healthcare.

## 2. Cardiovascular Disease in Women: Sex-Associated Differences

### 2.1. Clinical Setting and Risk-Assessment

Traditionally, cardiovascular disease has been considered purely male issues. However, epidemiological data reveal a very different reality: ischemic heart disease (IHD) has a higher incidence in women than in men; moreover, it is the leading cause of death and disability in the female sex in all Western countries [8,9]. 

Several studies suggest relevant gender differences in IHD regarding clinical risk factors, prognosis, diagnosis, and treatment [10,11,12].

Guidelines developed as early as 2011 by the American Heart Association (AHA), the European Atherosclerosis Society (EAS), and the European Society of Cardiology (ESC) specifically framed the risk profile and prevention interventions dedicated to the female gender, acknowledging, however, the low presence of women in cardiovascular risk studies [13]. In addition to the well-known traditional risk factors, divided into modifiable and non-modifiable, that women share with men, several studies have focused on the impact of emerging factors, considered more typically female, such as autoimmune disorders and/or psychosocial factors such as anxiety and depression. In addition to traditional and emerging factors, more recent studies have focused on gender-specific factors, which are exclusive to the female sex and are especially attributable to reproductive function (hypertension in pregnancy, pre-eclampsia, and gestational diabetes) and menopause [14,15].

In addition, it is known that women develop IHD with a latency of about 7–10 years compared with the male gender, thus being older and more frail, and being more likely to be simultaneously affected by other comorbidities and conditions that may mask their diagnosis but also affect their treatment choices. One possible explanation for this female latency in the incidence of IHD is due to the fact that during the childbearing years, women are protected by the estrogen umbrella [16].

Further gender differences concern the pathophysiological mechanisms related to the process of atherosclerosis. The latter, in fact, is more recent in women, resulting in less development of collateral circles [17].

In addition, women with IHD not only present more frequently than their male counterparts with normal or significantly unobstructed epicardial arteries, but they are also more prone to complications, acute and/or subacute, such as plaque erosion, spontaneous coronary artery tree dissections, stress cardiomyopathy, and heart rupture after acute infarction [11].

### 2.2. Diagnostic Management

As regard to diagnosis, on the other hand, this is very often missed and/or delayed in the female population. Different clinical manifestations mainly contribute to a very often missed and/or delayed diagnosis: in fact, the female gender does not complain of typical acute chest pain radiating to the left arm but more frequently manifests “atypical” symptoms, such as back pain, irradiation to the jaw, nausea and/or vomiting, dyspnea, palpitations, dizziness, fatigue, lack of appetite, and episodes of syncope [18]. As a result, many women wait longer before calling for help, resulting in higher pre-hospital and intra-hospital mortality and an increased risk of developing acute and sub-acute complications [19,20].

Additional factors impacting delayed diagnosis are a misperception of cardiovascular risk in the female sex by healthcare providers as well as lower reliability of diagnostic tests [21,22,23].

### 2.3. Cardio-CT and Clinical Setting

Cardio-CT (CTA) is a noninvasive method indicated in low-to-intermediate-risk patients with acute chest pain when all other investigations performed have not provided conclusive results, in patients with suspected or chronic ischemic heart disease and typical and/or atypical anginal symptoms, as well as in the follow-up of stent and bypass revascularization procedures to assess patency [24,25].

Quantification of coronary artery calcium (CAC), introduced in 1990 by Agatston et al., is a primary step in the cardio-CT study; this parameter is still widely used as a negative predictive parameter (nearly 100%) for objectively quantifying cardiovascular risk, especially for those patients at intermediate risk for further cardiovascular risk stratification [26].

However, it is known in the literature that coronary calcium quantification underestimates future cardiovascular risk in the female population [27,28].

**Figure 1:**
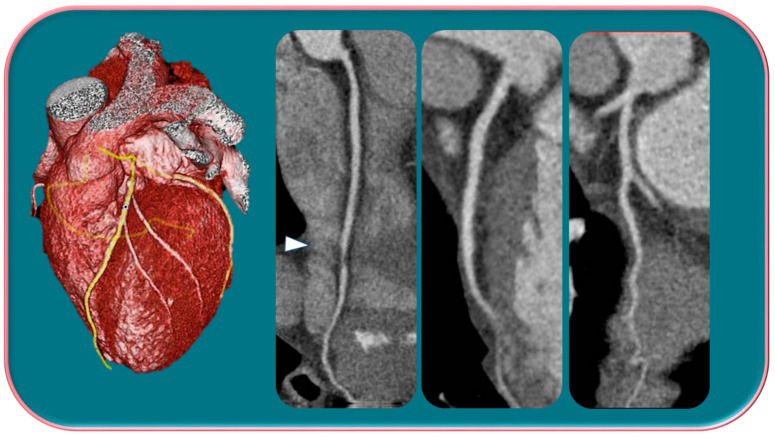
Male, 39-years-old, patients with history of dyslipidemia, type-1 obesity, and higher level of stress at work. The patient reported a non-anginal chest pain for which an ergometric ECG stress test was performed, and the results were negative. According to the Diamond–Forrester score, the patient was first categorized as a low pre-test probability risk of having CAD. To correctly rule-out CAD, the patients underwent CCTA examination, which showed a severe non-calcific plaque in the distal right coronary artery (white arrowhead).

Kim et al. [29] in a study evaluated gender differences regarding the distribution and prevalence of CAC, its relationship to clinical risk factors, and the effect of a high calcium score (≥100) on subsequent initiation of medical therapy by evaluating a court of 542 individuals, of whom 279 were female and 263 were male. The data demonstrated an increasing CAC score with age, regardless of sex. However, women showed significantly lower CAC scores than men in the same age group. The lower CAC scores in women are likely in part indicative of the fact that women have a greater amount of non-calcifying plaque, so-called low-attenuation plaque, than men, indicating that lower CAC scores may actually be related to higher-risk plaque. However, there are also cases, although less frequent, of men with low-attenuation atheromasic plaque (Figure 1). Another interesting finding emerged from the correlation between CAC and clinical risk factors: for both sexes, age had a similar effect on CAC score (OR 1.11 and 1.12, respectively); however, in men, obesity showed a significant effect on CAC score (OR 2.16), while in women, smoking showed a significant effect on CAC score (OR 4.27). This result clearly demonstrated that the traditional clinical risk factors and which women, therefore, share with men actually have different clinical impact in the two genders. Finally, it was investigated how the CAC score result affected the further management of men vs. women with a CAC score ≥100 when adjusted for concurrent coronary CTA findings (moderate to severe lesions vs. non-obstructive lesions) in initiating aspirin and statin therapy [29].

Kim et al. [29], moreover, reported that, while, for patients with moderate-severe stenosis (>50%) found at CTA, no gender difference was observed in initiation of therapy, strong differences were shown in patients with non-obstructive atherosclerotic lesions. Aspirin and statin use increased in both sexes but men with non-obstructive coronary artery disease (NOCAD) were significantly more likely to take preventive medications than women. In particular, among men with NOCAD, 68.2% were on aspirin and 86.4% were on statin after the CTA, which is a significant increase from 18.2% and 36.4% before the test, respectively. In contrast, among women with non-obstructive lesions, only 27.3% were on aspirin and 45.5% on statin after the test, compared to 18.2% each before the test [29].

In another study, Arslan et al. [30] showed that women had a lower incidence of obstructive CAD at CTA, underwent fewer revascularization procedures, and were hospitalized less frequently than men. In addition, in this same study, when comparing both diagnostic groups by sex, it was found that women underwent fewer outpatient examinations when early CTA was used in the emergency evaluation of suspected acute coronary syndrome. The lower burden of obstructive CAD in women detected at CTA was found to be a possible explanation as it reassured healthcare providers not to perform additional outpatient examinations compared with the male counterpart.

Although patients with angina without obstructive CAD have a better prognosis than those with obstructive epicardial CAD, they are still at higher risk of developing cardiovascular disease than the baseline population. Therefore, physicians need to be vigilant in patients with recurrent angina without obstructive epicardial CAD, especially in women, and initiate further evaluation such as high-sensitivity troponin, which are new cardiac biomarkers that can detect myocardial damage quickly and very accurately, or further diagnostic investigations in suspecting microvascular disease and treat accordingly.

In recent years, technological innovation has caused CTA to undergo enormous implementation as well [31]. Thanks to the development of innovative software using automated quantification techniques, there is now the possibility to identify the total plaque volume and its characteristics, i.e., to highlight low-attenuation plaque (<30 HU), the so-called high-risk plaque that is the strongest predictor of future myocardial infarction regardless of CAC, clinical risk factors or severity of stenosis, the percent volume of the atheroma (the proportion of the total volume of the vessel wall occupied by atherosclerotic plaque), as well as the volume of plaque indexed to the size of the coronary artery or patient. Anatomically, women have smaller hearts and vessels even after adjustment for body size, and clearly, this can all influence the choice and accuracy of diagnostic tests [32].

Finally, additional approaches such as fractional flow reserve that allow us a hemodynamic assessment of coronary artery injury, myocardial stress perfusion in CT, and future research exploring techniques such as shear stress, left ventricular mass assessment, and strain, are further implementing the role of CTA, and it is hoped that, within a short time, they can be introduced into daily clinical practice [33,34,35,36,37,38,39].

Some most recent studies investigated the impact of gender differences on the diagnostic performance of machine-learning-based coronary CT angiography (CCTA)-derived fractional flow reserve (CT-FFRML) for the detection of lesion-specific ischemia [40,41,42].

Specifically, one article [43] compared machine-learning FFRCT between men and women to determine whether there are gender differences in the distribution of ML-FFRCT according to the degree of coronary stenosis and specific coronary vessel involvement, as well as whether there is any gender difference in the prognostic significance of ML-FFRCT in relation to incident or MI cardiovascular outcomes. In that study, in a high-risk cohort of symptomatic patients undergoing CCTA and SPECT, the degree of coronary atherosclerotic disease was lower and ML-FFRCT was higher in women than in men. However, no significant association was found between FFRCT and incident mortality or myocardial infarction (MI), and there is no evidence that the prognostic value of ML-FFRCT differs by sex.

### 2.4. Cardio-MRI and Clinical Setting

CMR is a high-spatial- and -temporal-resolution, noninvasive imaging modality that offers the added benefit of no exposure to ionizing radiation, so it is particularly advantageous in women of childbearing age. CMR also provides excellent assessment of myocardial structure and function, as well as signs of inflammation, ischemia, and myocardial viability [44,45,46,47,48].

**Figure 2:**
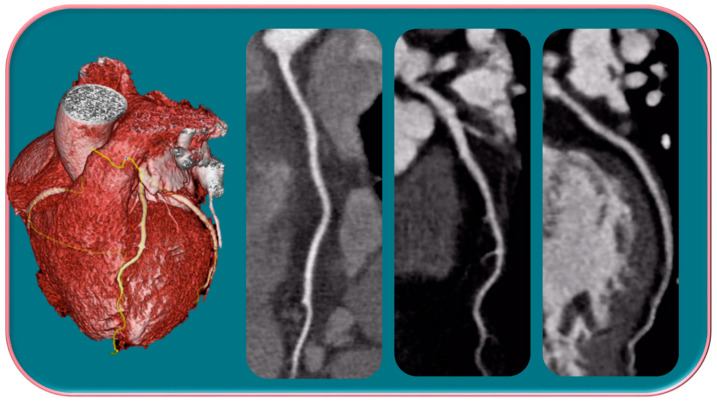
Female, 65-years-old, with typical angina, treated for hypertension and dyslipidemia. The patient was first categorized as intermediate-PPT risk of having CAD, for which CCTA was performed. The CCTA exam showed a regular coronary tree. Different trials have demonstrated that women show more frequent anginal symptoms than men, often with a higher risk profile, although a lower overall burden of CAD, a higher prevalence of NOCAD, and a frequent insufficient ischemia have been highlighted.

Several studies have shown that there is an over-representation of women with myocardial infarction with unobstructed coronary arteries (MINOCA) (Figure 2) [49,50,51].

The mechanisms underlying MINOCA most commonly observed in women include coronary microvascular dysfunction and plaque erosions. Identification of the underlying etiology of MINOCA is, therefore, critical for proper risk stratification and with regard to treatment [52,53].

Several recent studies are focusing on the myocardial perfusion reserve index obtained by first-pass perfusion CMR imaging with vasodilator stress that can detect coronary microvascular disease in a manner quite similar to invasive coronary reactivity testing [54,55].

Another question that CMR can answer is whether coronary microvascular dysfunction is related to or even causes myocardial tissue damage [56].

In conclusion, we have seen how important it is to know the gender aspects in terms of the clinical expression of the disease, which is essential to diversify the diagnostic work-up. Therefore, we believe it is essential that future guidelines take into account gender differences regarding diagnostic investigations in order to improve patient management and prognosis. However, despite significant advances in noninvasive cardiac imaging, more evidence is needed to support a gender-based first-line diagnostic work-up.

## 3. Sex Differences in Interventional Radiology

Several articles in the literature have focused on gender differences regarding the management and treatment of abdominal aortic aneurysms (AAAs).

AAAs are 4–6 times more common in men than in women [57]. However, the female sex is associated with a higher risk of rupture and worse peri-operative outcomes after AAA repair [58]. Although the etiology of these differences is not fully elucidated, the influences of sex hormones, more complex anatomy, more graft-related complications, and a higher incidence of undiagnosed cardiovascular disease have been suggested as potential causes [59].

In particular, one study [60] showed that women presenting in the emergency department with an AAA were less likely to undergo repair than men. Although part of this finding could be explained by differences in age and comorbidities, the differences persisted even after case-mix adjustment.

Previous studies have also shown that the female sex is independently associated with a faster rate of AAA growth and tends to go into rupture with smaller aortic size than its male counterpart [61,62]. Finally, some studies have shown that estrogen may play a protective role in AAA development, while testosterone has a negative effect on wall stress [59,63,64].

A recent comparative study compared open surgery with endovascular treatment (EVAR) of complex abdominal aneurysms [65]. Abdominal aneurysms involving the renal and visceral segments of the aorta are also known as complex AAAs [66].

In the above study [65], women showed higher rates of complications and mortality following EVAR than the male gender. However, after open repair, the peri-operative mortality and major complication rates were similar between female and male patients. Thus, the peri-operative mortality benefit of EVAR over open repair in male patients was not found in female patients.

It is also hypothesized that the female sex has a greater negative prognostic impact on the outcomes of endovascular treatments of complicated AAAs than their male counterparts, due to the more challenging procedures, performed using stiffer devices in subjects with smaller vessels and more complex anatomy. The introduction of new endovascular repair strategies such as fenestrated and branched endografts, or chimney and snorkel techniques, have made endovascular repair of these complex aneurysms possible with good results [67]. However, the promising results for complex EVARs may not be applicable to the female population, as females are typically underrepresented in these studies and are less likely to meet the necessary anatomical criteria for transplantation than male patients.

Gender differences regarding the onset, clinical presentation, development of complications, and treatment have also emerged in abdominal aortic dissections (AAD), although little evidence exists.

Takahashi et al. [68] analyzed data from 2372 (695 women, 29.3%) patients with type B AAD enrolled in the Tokyo Acute Super-Network Registry and concluded that women were older than men and presented to the emergency department later. Women also had a higher percentage of intramural hematoma (63.7% vs. 53.7%, *p* < 0.001), underwent more conservative treatment (90.9% vs. 86.3%, *p* = 0.002), and had a higher in-hospital mortality (5.3% vs. 2.7%, *p* = 0.002).

However, given the scarce studies in the literature, more data are needed to identify gender-specific determinants regarding functional outcomes of endovascular treatments.

## 4. Sex Differences in Respiratory Pathologies

### 4.1. Clinical Setting

The literature has reported important gender differences regarding the anatomy and physiology of the respiratory system.

The development of male and female lungs is a process that is highly regulated and controlled by genetic, epigenetic, environmental, as well as hormonal factors.

During the fetal period, male lung maturation is known to be usually delayed compared with female lung maturation [69,70]. This impacts lung surfactant production, which begins later in the male lung than in the female lung, resulting in a greater likelihood for male infants to develop respiratory distress syndrome (RDS).

Hormonal changes during development and puberty, as well as physiological events such as pregnancy and menopause, also appear to affect lung function by influencing the onset, severity, exacerbation, and mortality rates of many lung diseases common to men and women. Indeed, while estrogen appears to exert a beneficial effect early in life by promoting lung development and maturation, androgens, in contrast, appear to exert an opposite effect. This highly regulated process is likely to be reversed during adult life with regard to certain diseases such as severe asthma, where improvement is observed with increasing androgen levels, while fluctuations in female hormones during the menstrual cycle promote their exacerbation [71]. Indeed, it is well known that women exhibit cyclic symptoms in relation to the menstrual cycle, with higher measures of lung function during the luteal phase than during other phases of the cycle [72,73].

Respiratory infections remain a major cause of morbidity and mortality in all age groups. A complex interaction between genetic factors, sex hormones, immunity, anatomical differences, and sociocultural and behavioral factors between men and women could explain the observed sex differences in the rates and severity of lung infections, making the radiological pictures more complex.

### 4.2. Coronavirus Disease 2019

The impact of sex and gender in the incidence and severity of coronavirus disease 2019 (COVID-19) has been widely discussed in the literature [74,75,76]. In almost all countries, a significant male predominance in morbidity and mortality from COVID-19 has been reported, suggesting a biological mechanism [77,78,79].

To understand the greater severity regarding SARS-CoV-2 in men than in their female counterparts, it is possible to look at the mechanisms of immune system activation: in men, in fact, a greater severity in prognosis appears to be related to increased plasma levels of innate immune system cytokines, including IL-8 and IL-18. In contrast, a lower severity in women corresponded to a greater activation of T cells [80,81,82].

In addition, sex hormones also appear to play a role in SARS-CoV-2: testosterone is associated with a worse prognosis in relation to its possible role in suppressing the inflammatory process [83].

Regarding imaging, some studies have focused on the prognostic role of chest radiography (CXR) and computed tomography (CT) [84,85,86,87,88].

An Italian study [89] proposed an experimental semiautomated scoring system for CXR aimed at quantifying the severity and progression of pulmonary abnormalities in COVID-19 pneumonia. The CXR score was then retrospectively correlated with age and sex. The results showed that males aged 50 years or older and females aged 80 years or older had a higher CRX score (>8), indicative of severe pathology. These results are crucial as they allow us to identify high-risk patients in order to evaluate diagnostic-therapeutic strategies declined according to gender.

Moradi et al. [90], in another study, instead validated the role of chest CT in the management of COVID-19. In this study, the correlation between sex and disease severity based on CT findings was demonstrated. One hundred fifteen patients (64.3% [74/115] men), with a median age of 57 years (age range, 21–89), were enrolled, of whom patients were admitted to the intensive care unit, and 30 died during hospitalization. Seventy-seven percent (37/48) of patients with poor prognosis were male. The peripheral distribution of opacities was more common in men than in women. When grouped according to an age threshold of 60 years, women in the oldest group, and thus older than 60 years, had a peri-bronchovascular distribution pattern, while younger men showed an anterior distribution of opacities. Women younger than 60 years had significantly lower severity scores (CT-scores) (7.5 ± 6.8). A possible explanation for the lower CT-scores in women younger than 60 years could lie in estrogen immuno-protection mechanisms responsible for a subsequent decrease in viral genome transcription with an associated increase in immune clearance as a result of α-receptor activation. Androgens, in contrast, have the opposite effect through androgen receptor signaling in viral infections. In addition, the study showed that the use of severity CT scores can predict short-term prognosis in both men and women younger than 60 years of age and, especially in the latter group, with high sensitivity and specificity. In fact, receiver operating characteristic (ROC) curve analysis demonstrated a CT-score cut-off of 14.5 to have 100% sensitivity and 91.9% specificity for predicting poor prognosis in women younger than 60 years. The pandemic has also necessitated the development of models to support clinical practice using artificial intelligence (AI) techniques [91,92,93,94]. Currently, the supporting models reported in the literature regarding COVID-19 have mainly focused on epidemic trends, early screening, and CT diagnosis as well as prognosis [95,96,97,98,99,100].

However, there are not yet studies in the literature investigating the role of AI in gender differences in COVID-19.

### 4.3. Cystic Fibrosis and Idiopathic Pulmonary Fibrosis

Gender differences also exist in additional respiratory diseases such as cystic fibrosis (CF) and/or idiopathic pulmonary fibrosis (IPF).

CF is an autosomal recessive disease resulting from malfunction of the protein CFTR, a transmembrane protein that leads to dehydration and viscous secretions in the lumina of affected organs. The main causes of morbidity and mortality are respiratory involvement, progressive pathogenic infection by specific bacteria such as Pseudomonas aeruginosa, and the host inflammatory response, which results in poorer pulmonary function and altered pulmonary architecture [101,102].

**Figure 3:**
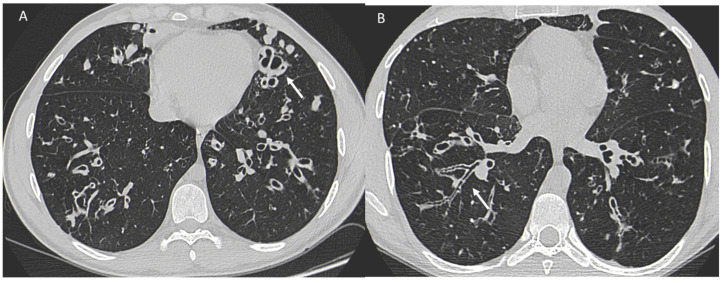
A woman and a man with CF with different and higher anatomo-structural involvement at HRCT in women with CF compared with men. (**A**) Diffuse cystic-varicoid bronchiectasis, with diffuse wall thickening and mucous plug (white arrow) in a young woman patient with cystic fibrosis. (**B**) Bronchiectasis with thickened walls (white arrow), especially in the medium-lower pulmonary lobes in a male patient with cystic fibrosis.

One study [103] investigated the survival of women with CF compared with men and showed that survival in the female population with CF is lower than that of men by about 5 years. Underlying this discrepancy is a different and higher anatomic-structural involvement on high-resolution chest CT (HRCT) in women with CF compared with men (Figure 3). Indeed, higher scores were recorded in women regarding the extent of bronchiectasis, involvement of bronchial branches, and severity of bronchiectasis, thus illustrating a virtually generalized involvement of the bronchial tree, as well as regarding air entrapment. This resulted in more frequent exacerbations, worse functional and nutritional outcomes, deterioration of quality of life, and greater structural damage in the female population.

IPF, on the other hand, is a male-dominated disease. In international cohorts, males account for about 70% of all IPF cases. A recent study found that clinical physicians rarely assign the diagnosis of IPF to women and that gender is the most discriminating pre-test diagnostic probability criterion according to them [103].

**Figure 4:**
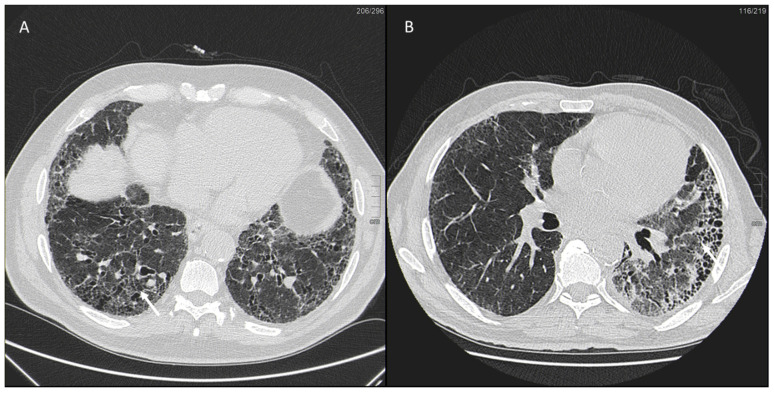
(**A**) Diffuse ground glass opacities with reticular thickening of the subpleural interstitium (white arrow), together with traction bronchiectasis in a case of pulmonary fibrosis with UIP pattern and smoking-related interstitial lung disease (ILD) in a female smoker. (**B**) A typical UIP pattern in a male smoker with idiopathic pulmonary fibrosis, together with diffuse ground glass opacities (white arrow) during an acute exacerbation.

IPF is a chronic, irreversible, disabling disease with a fatal outcome characterized by a progressive decline in lung function. It is associated with a radiological pattern of usual interstitial pneumonia (UIP). In the literature, women have shown less fibrotic alterations than their male counterparts at HRTC (Figure 4).

A French multicenter prospective study [104] explored gender differences in an IPF cohort over a 5-year follow-up period. The cohort included 51 (22%) females and 185 (78%) males with a mean age at diagnosis of 70.1 ± 9.20 and 67.4 ± 10.9 years, respectively. At presentation, honeycombing and emphysema at HRCT were less common in females: (n = 40 (78.4%) vs. n = 167 (90.3%), *p* = 0.041) and (n = 6 (11.8%) vs. n = 48 (25.9%), *p* = 0.029), respectively. Fewer women than men also underwent transplantation during follow-up (n = 1 (1.96%) vs. n = 20 (10.8%), *p* = 0.039).

This is consistent with an earlier report that suggested that surgical lung biopsy for the diagnosis of IPF was required more frequently in women than in men [105].

In addition, although women appear to have less advanced disease at the time of diagnosis, perhaps due to less exposure than men, disease progression and overall survival remain comparable regardless of sex [104,106].

### 4.4. Chronic Obstructive Pulmonary Disease

Finally, it has been shown that significant gender differences in susceptibility, severity, and response to therapy also affect patients with chronic obstructive pulmonary disease (COPD) [107,108,109].

COPD until recently was considered a disease of elderly men who were smokers [110].

**Figure 5:**
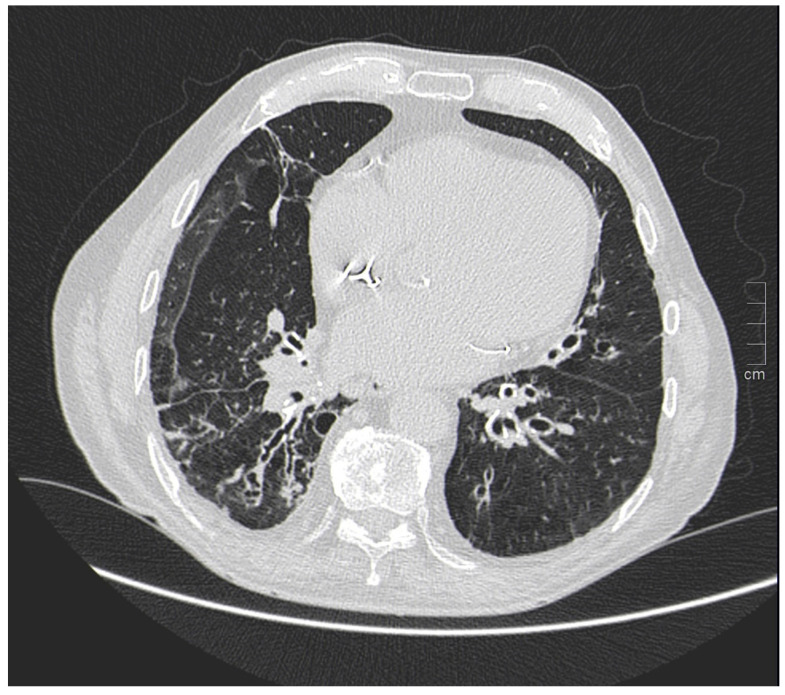
Arrow shows diffuse thickening of the bronchial wall in a former-smoker woman with COPD.

However, as our knowledge evolves, COPD is increasingly considered a disease with a strong impact on women. Behavioral, environmental, sociocultural, and clinical factors have likely contributed to the increased prevalence of COPD in women and, in particular, to the increased prevalence of smoking among women in developed countries (Figure 5). In addition, other risk factors have contributed albeit to a lesser extent to the increased incidence of COPD in women such as occupational exposures and respiratory infections, such as tuberculosis [111].

Structural changes in the lungs in airway disease and emphysema also differ in males and females.

Advances in computed tomography (CT) have allowed for the quantitative assessment of the extent of airway disease and emphysema.

In a recent study, Gu et al. [112] hypothesized that there are gender differences in airway functional and pathological changes that can be identified by HRCT and pulmonary function tests (PFTs). Based on HRCT results, more males were classified as phenotype M, indicating the presence of discrete emphysema associated with bronchial wall thickening, while women more frequently showed phenotype A, indicating reduced or absent emphysema not associated with bronchial wall thickening. Women also showed less airway wall thickening than their male counterparts, although this change had no statistical significance (χ^2^ = 0.163, *p* = 0.92). In addition, advances in CT imaging have enabled more detailed analysis of airway dimensions of patients with COPD. It has been suggested that CT measurements have the potential to represent histologic airway size changes in relation to the degree of small airway remodeling brought about by pathology [113].

In contrast, another study [114] showed that lumen area, inner diameter, and wall thickness were smaller in women than in men in all airways measured. Airway dimensions were measured using automated quantitative software designed to label and quantify the bronchial tree. However, in the same study, women had a higher wall area percentage (WA%), i.e., WA/[WA + lumen area] ×100, in sub-segmental and sub-sub-segmental bronchi. That study showed that female smokers had higher WA%, but smaller luminal area, inner diameter, and airway thickness measured by CT at anatomically matched sites (sub-segmental and sub-sub-segmental bronchi) than male smokers. This difference could explain, in part, gender differences in the prevalence of COPD and airflow limitation.

This finding is in agreement with a further study [113]. A broader understanding on sex differences in the pathophysiology of COPD may help in designing sex-based diagnostic and treatment strategies.

Finally, few studies have focused on gender differences regarding the diameter, length, area, and branching angles of the trachea and bronchi, such as subcarinal angles (SCA) and inter-bronchial angles (IBA), as well as the identification of the various types of trachea using CT images in order to guide bronchoscopy procedures and endobronchial stent applications. CT has again proven to be a useful technique for morphologic assessment of the bronchial tree; however, further research is needed in this area as well [115,116].

## 5. Sex Differences in Oncology

Sex is a key biological factor influencing the development of many types of cancer. Indeed, there are also considerable differences in the field of oncology between male and female subpopulations in terms of incidence, prognosis, mortality, and response to treatment.

Internal factors (genetic, epigenetic, and hormonal) and external factors such as sex-related social behaviors (smoking and alcohol consumption) offer an albeit complex explanation for these differences. Knowing and valuing gender differences in this area as well is necessary, as it makes it possible to ensure the best care for each individual through the personalization of treatment and the assurance of gender-defined diagnostic and therapeutic pathways, as well as to promote gender-specific prevention initiatives through the identification of gender-specific risk factors. Here, we examine gender differences regarding the incidence, diagnosis, prognosis, and treatment of some common cancers and their related clinical implications.

## 6. Lung Cancer

Lung cancer (LC) remains a significant health problem worldwide, for both men and women, and is the leading cause of cancer death in many countries [117].

Although age-adjusted LC incidence rates have in past years been higher in men than in women to date, there is a significant increase in incidence in women [118].

Cigarette smoking remains the most important risk factor, and the increase in tobacco use in women in many parts of the world will further exacerbate its incidence and mortality in the female population [119]. However, tobacco exposure alone does not explain the whole problem.

Numerous data find that there are strong gender differences in the biology of LC. As evidence of this, women with LC are younger than men and smoke less intensively. In addition, over-representation of adenocarcinoma and small cell lung cancer (SCLC) has been observed in women [120]. Of particular interest also is the increased incidence of LC in women who never smoked and had the least exposure to occupational carcinogens; this suggests that other mechanisms may play a role in the increased incidence in the female population. One study [121] summarized all factors that may play a role in the pathogenesis of lung cancer in women. Estrogen receptors (ERs) and, in particular, ERb have, for example, been demonstrated in human non-small-cell lung carcinoma (NSCLC) cell lines and found to be overexpressed compared with normal lung tissue. In addition, progesterone has been shown to inhibit cell proliferation by inducing apoptosis in NSCLC. Clearly, this finding, if validated, could potentially be exploited in the design of new personalized therapies.

In contrast, it is known in the literature that hormone replacement therapy increases the number of lung cancer deaths, particularly in NSCLC. These findings should, therefore, be incorporated into discussions of the risk–benefit ratio in women considering combined hormone therapy and particularly in those with a high risk of developing LC [122].

However, in the face of these less than comforting data on the risk of developing LC, women show better short- and long-term survival than men. In fact, some studies have shown that women, if treated early, have better survival after surgical resection as well as a greater response to chemo- and radiotherapy as well as immunotherapy [123,124].

The current guidelines for screening tests for LC by low-dose computed tomography despite the benefits to the female population in relation to the ability to make an early diagnosis are currently targeting individuals with a history of smoking and likely under-represent the additional risk factors for LC in the female population [125,126,127].

Moreover, to date, there are still conflicting results regarding the actual benefits of CT screening not only because of the investigation itself, which requires exposure to ionizing radiation, but also because of the high number of false positives that could result in further increasing the number of additional invasive surgeries required such as bronchoscopy, percutaneous biopsy, thoracoscopy, and thoracotomy and, consequently, mortality and morbidity [128].

Finally, some studies are focusing on the possibility of applying radiomics to daily clinical practice in order to improve not only the detection of lung lesions but also the ability to distinguish malignant from benign lesions, characterize their histology, stage, and genotype, as well as the response to treatments and the possibility of applying the models to lung cancer screening in order to overcome the limitation of over-diagnosis of indeterminate nodules [129,130,131,132,133,134].

## 7. Colorectal Cancer

Colorectal cancer (CRC) is one of the most common causes of morbidity and mortality in both men and women [135,136].

**Figure 6:**
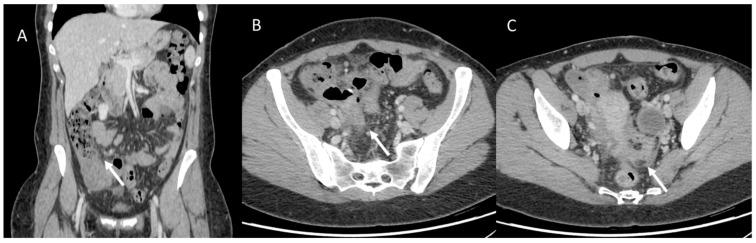
Woman with operated appendiceal tumor ((**A**): in coronal plane, arrow). CT assessment of peritoneal carcinomatosis ((**B,C**), arrow).

**Figure 7:**
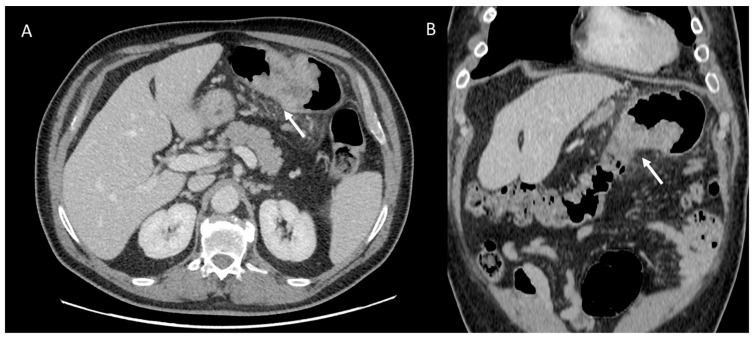
Man with transverse colon cancer (white arrows): in (**A**) (axial) and (**B**) (coronal) CT assessment.

However, gender disparities are still a poorly considered aspect of CRC management. Women have a higher risk of developing right (proximal) colon cancer, which is associated with a more aggressive form of neoplasm than descending colon cancer, which is more typically male (Figure 6 and Figure 7) [137]. Underlying these gender differences are different genetic polymorphisms, epigenetic and hormonal factors, as well as a number of modifiable environmental factors, including lifestyle [138].

In addition, proximal colon tumors, even advanced ones, are more often flat in contrast to distal colon tumors, which, on the other hand, tend to take on a polypoid morphology more often, so they are more easily distinguishable on colonoscopy. [139] In addition, women possess a longer transverse colon than men, resulting in a lower detection rate at colonoscopy [140]. One study [141] in fact showed that women had an overall decreased colonic volume, increased tortuosity and compactness, and lower sigmoid apex height on CTC compared to men (*p* < 0.0001). This finding could, therefore, explain the lower sensitivity of diagnostic tests resulting in delayed diagnosis. The sensitivity of the fecal occult blood test (iFOBT), one of the most commonly used CRC screening tests, was also found to be different according to gender [142].

Another fact not to be underestimated about CRC is that the incidence and mortality in women are delayed by at least 5 years compared with men. Therefore, the incidence and mortality of CRC in the population over 65 years old is higher in women than in men. In addition, the 5-year survival rate of CRC among women is lower than that of men, and this is particularly evident in women older than 70 years of age [143].

CRC screening offers an effective opportunity to prevent the disease; however, it is only targeted at a segment of the population (50–69 years old). [144] To date, there are also no gender-specific screening tools or guidelines.

A Norwegian randomized controlled trial [145] investigated the long-term effects of screening with sigmoidoscopy on incidence and mortality in CRC and provided decidedly surprising results. Screening was compared with “no screening” in a sample of nearly 100,000 subjects aged 54–64 years with no familiarity of CRC. CRC incidence and mortality were reduced in men, while there was little or no effect in women. One possible explanation for these results may be the latency of CRC incidence in the female population. Thus, it would be necessary to date to review the screening programs and possibly to extend them to the female population over 70 years of age for the above reasons.

Finally, clinical practice support models have also been developed for CRC using AI techniques [146,147,148]. However, their application in daily clinical practice especially with regard to gender differences is still rather immature.

## 8. Breast Cancer

Breast cancer (BC) is the most common global malignancy and the leading cause of cancer deaths [149].

BC is the most common malignancy in females but it is rare in males, representing 0.5% of all breast cancers [150]. However, the occurrence of male breast cancer (MBC) is increasing nowadays due to the aging population. Furthermore, although in female breast cancer (FBC), mortality rates have decreased over the past decades due to advances in diagnostic procedures and treatments, mortality rates in MBC have remained essentially constant or modestly increased [151].

Because MBC has a very low incidence, the literature, research, and clinical trials regarding the development of new treatment options have focused mainly on FBC. In addition, although knowledge about FBC can aid the diagnosis and treatment of MBC, it is known that the molecular and clinic-pathological features differ between male and female BC [152]. Genetic and hormonal factors must, therefore, be considered when discussing breast cancer in men and deciding on treatment options. Currently, there is no standard of care (SOC) for MBC and there is an unmet need for research regarding diagnostic and treatment options for this disease.

Advancing age, hormonal imbalances, radiation exposure, and a family history of breast cancer are the major risk factors for the development of male breast cancer. The most prominent risk factor for the development of male breast cancer is a mutation in the BRCA2 gene [153]. However, mutations in other DNA repair genes, such as CHEK2 and PALB2, are also associated with MBC [154,155].

In addition, epigenetic alterations are also implicated in cancer development and progression. Similar to FBC, promoter hypermethylation and some genes implicated in pathology have also been found in MBC. The difference is that many of the genes analyzed in the male population with a breast cancer predisposition have a reduced methylation frequency compared with FBC, which provides further differences between the development and progression of male and female BC [156].

Implicated also in the pathogenesis of male breast cancer is Klinefelter syndrome, which increases the risk of developing MBC by up to 50 times compared with unaffected men. This represents a rare genetic disease characterized by the presence of an extra X chromosome (XXY genotype) and is associated with testicular dysgenesis, gynecomastia, and androgen and estrogen balance alteration with increased endogenous estrogen production [157].

**Figure 8:**
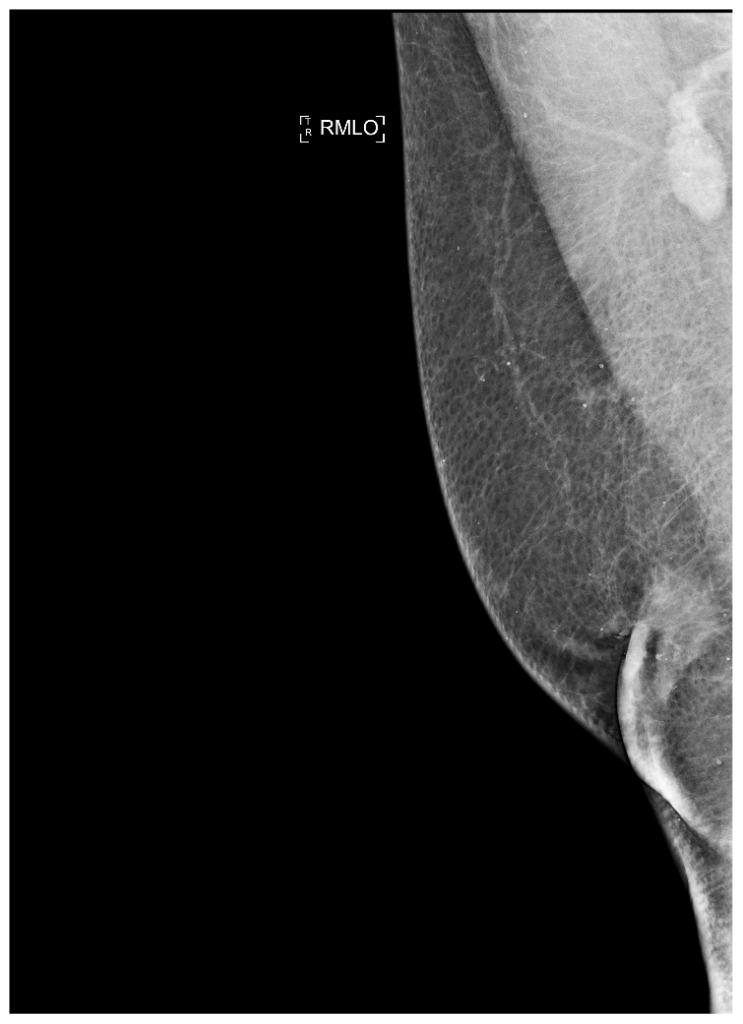
Man with retroareolar tumor and skin thickening.

Similarly, an increase in exogenous estrogen exposure, such as the estrogen treatment of prostate cancer and transgender hormone therapy, is responsible for an increase in risk of male BC (Figure 8) [158].

Endogenous causes of hyperoestrogenization in men, including obesity, cirrhosis, mumps orchitis, or testicular lesions, have also been associated with an increased risk of MBC.

It is also interesting to note that invasive lobular BC is very rare in men due to the poor representation of terminal mammary lobules, except for those who are exposed to increased estrogen from endogenous or exogenous sources [159].

Most men develop invasive ductal carcinomas with a latency of about 5–10 years from FBC [160,161].

A recent article by Greif et al. [150] highlighted differences in overall survival (OS) in men and women with BC. OS was highly significant (p0.0001): 83% 5-year OS for women with breast cancer (median survival 129 months) versus 74% for men (median survival 101 months). Women also had better 5-year OS (*p* = 0.0001) for stage 0 (94% vs. 90%), stage I (90% vs. 87%), and stage II (82% vs. 74%). There were no differences in 5-year OS for stage III (56.9 vs. 56.5 %, *p* = 0.99) or stage IV (19 vs. 16 %, *p* = 0.20).

In addition, MBC is more likely to express receptors for estrogen (OR) and progesterone than FBC. In contrast, tumors positive for human epidermal growth factor receptor 2 (HER2) are considered quite rare in males (1.7 percent vs. 6–12 percent in the female counterpart) [162].

Finally, MBC is often diagnosed at a more advanced stage than FBC, and this may often be related to a diagnostic delay [163].

For men, in fact, there is no specific screening with mammography to identify the tumor in the early stages, because MBC is, to date, considered too rare to periodically screen the entire population. Hence, diagnosis requires a high index of suspicion, mainly because of the low awareness of this cancer in the male population. Despite this, males who are sons or brothers of women carrying the BRCA1 and BRCA2 mutations should have genetic testing, because if they are found to be carriers of the mutated gene, they too would be at increased risk of developing cancer, so close follow-up would be necessary [164,165].

BC diagnosis in men to date is similar to its female counterpart. It is based, first after an adequate personal and family history, on the clinical examination, which may be followed by an in-depth diagnostic examination. In this case, ultrasonography and mammography are also used for men to visualize the structure of the breast, possibly supplemented by cytological analysis of the fluid that, in some cases, leaks from the nipple. However, the gold-standard diagnostic test is biopsy. Once a diagnosis is made, it is necessary to determine certain characteristics of the cancer cells such as the presence or absence of receptors for hormones (estrogen and progesterone) or levels of the HER2neu protein, which are very important to guide the clinician in choosing the most effective treatment [166,167,168].

Finally, CT, positron emission tomography (PET), and bone scintigraphy represent the most commonly used tests to identify the presence of distant metastases [169,170,171].

Given the frequent diagnostic delay involving MBC at an early stage, it might be useful to define a specific protocol based on the literature and future perspectives, which can define the proper use of imaging in the case of men with BC. The committee recommends mammography or digital breast tomosynthesis (DBT), especially in those cases of architectural distortion or in cases of gynecomastia, in men 25 years of age or older if symptomatic or if clinical examination suggests the presence of BC [172,173,174].

Ultrasound correlation, according to a recent paper published by Tari et al. [175], strengthens the diagnostic pathway, while MRI possesses the same indications in men as breast cancer in women [176].

Recent studies have also explored the possible use of radiomics in breast pathology for the purpose of distinguishing benign from malignant lesions, depicting tumor biology, differentiating between early and advanced stages, predicting response to treatment, as well as determining the risk of recurrence [177,178,179].

A model based on radiomic analysis with the traditional imaging features of mammography to distinguish benign from malignant lesions in men was proposed by Huang et al. [180] They also developed and validated an imaging-radiomic nomogram for physicians to determine the risk of breast cancer for each male individual.

Finally, the inclusion of transgender people in clinical research and the identification of specific diagnostic pathways is another goal to be achieved.

A recent study [181] showed that transgender women (male sex assigned at birth, female gender identity) are at greater risk of breast cancer than cisgender men, while transgender men (female sex assigned at birth, male gender identity) are at less risk than cisgender women.

This showed that a relatively short exposure to hormonal therapy is responsible for an increased risk of breast cancer in trans women.

Transgender people resort to gender-confirming surgery and/or drug therapies, that is, treatments aimed at aligning physical appearance with their gender identity. Pharmacological therapies rely on the use of hormones such as estrogen or androgens, which, while fairly safe to date, could result in a long-term change in the risk of developing cancer [182]. Because the risk of breast cancer in trans women is increased in relation to a relatively short exposure of hormone treatment, it would be appropriate for future studies to investigate in more detail the cause of breast cancer in transgender people on hormone treatment.

Few guidelines currently exist for cancer screening and management for this sub-population that suggest that trans women and trans men who have not undergone mastectomy should undergo biennial mammography screening starting at age 50 years as well as even if they have been undergoing hormone treatment for more than five years, while monitoring trans men with mammography after subcutaneous mastectomy is not considered effective, because of minimal residual breast tissue [183,184,185].

However, to date, there are still many gaps in the literature regarding the lack of data in the transgender population and long-term follow-up.

## 9. Precision Medicine: Radiomics and Radiogenomics—The New Frontier of Imaging

A goal of modern medicine that also involves the branch of radiology is represented by “precision medicine,” the purpose of which is to personalize the diagnostic therapeutic pathway based on the specific characteristics of the patient and their disease, making it a tailored and, above all, more efficient pathway. This clearly has an impact not only from a clinical-medical point of view and on the patient’s quality of life but also from a socioeconomic point of view.

The natural evolution of precision medicine is the development of tools to support daily clinical practice.

It is no coincidence that, precisely in this historical period, there is this need for the collection of information, “the so-called big data“, supported also by the development of artificial intelligence techniques also in the field of radiology [186,187,188].

Here, in this context, radiomics is rapidly emerging as a technology to support personalized medicine [189,190,191,192].

Radiomics is a developing field, especially in oncology, that allows through quantitative analysis to characterize tissues noninvasively and, because tumors are heterogeneous in their volume and change over time, to monitor changes induced over time by therapies (delta radiomics) [193,194,195,196,197,198].

Diagnostic images through radiomics are then converted into numerical data (so-called “big data”) that will then be subsequently analyzed by dedicated computational tools using artificial intelligence methods [199,200].

With such techniques, it is also possible to derive information directly from the images on the molecular and genomic characteristics of the tumor (radiogenomics) with the ultimate intent of extracting indications on aggressiveness, prognosis, and therapeutic response [77,133,201,202,203,204,205,206,207,208,209,210,211,212,213,214,215,216,217,218,219,220,221,222,223,224,225,226,227,228,229,230,231,232].

The integration of quantitative data extracted from images with genomic expression and patient clinical data obtained and stored through the development of the structured report and the creation of databases will allow the creation of models to support clinical practice, which, it is hoped, will in the near future improve diagnostic accuracy and prognostic power, as well as ensure a more advanced level of precision medicine, which is essential for the enhancement of gender differences.

## Data Availability

All data are reported in the manuscript.
